# Unveiling the Power of Flax Lignans: From Plant Biosynthesis to Human Health Benefits

**DOI:** 10.3390/nu16203520

**Published:** 2024-10-17

**Authors:** Zhan Gao, Qinglei Cao, Zhongyuan Deng

**Affiliations:** 1 School of Physical Education and Training, Capital University of Physical Education and Sports, Beijing 100191, China; 2 Department of Physical Education, University of Science and Technology Beijing, Beijing 100083, China; cqinglei72@163.com (Q.C.); dzy@zzu.edu.cn (Z.D.); 3 School of Agricultural Sciences, Zhengzhou University, Zhengzhou 450001, China

**Keywords:** secoisolariciresinol, lignan synthesis, enterodiol, enterolactone, lignan metabolism

## Abstract

Background: Flax (*Linum usitatissimum* L.) is the richest plant source of lignin secondary metabolites. Lignans from flax have been applied in the fields of food, medicine, and health due to their significant physiological activities. The most abundant lignan is secoisolariciresinol, which exists in a glycosylated form in plants. Results: After ingestion, it is converted by human intestinal flora into enterodiol and enterolactone, which both have physiological roles. Here, the basic structures, contents, synthesis, regulatory, and metabolic pathways, as well as extraction and isolation methods, of flax lignans were reviewed. Additionally, the physiological activity-related mechanisms and their impacts on human health, from the biosynthesis of lignans in plants to the physiological activity effects observed in animal metabolites, were examined. Conclusions: The review elucidates that lignans, as phenolic compounds, not only function as active substances in plants but also offer significant nutritional values and health benefits when flax is consumed.

## 1. Introduction

Flax (*Linum usitatissimum* L.) is an annual or perennial self-pollinating herb [1]. Flax is one of the earliest cultivated fiber crops, with cultivation beginning as early as the Pre-Pottery Neolithic period (around 8500 BC). Flax, belonging to the genus *Linum*, originated mainly in the Near East and the Mediterranean coast. It has been cultivated primarily for food, fiber, and oil. Owing to its rich oil content, it is often referred to as ‘oil flax’ and is highly valued. Additionally, flaxseed contains several active ingredients that have important physiological functions, resulting in increased scientific attention in recent years. These include α-linolenic acid (ALA), linoleic acid, lignans, and linseed gum [2,3]. Flaxseed oil content ranges from 35% to 45%, of which 45% to 52% is ALA, an omega-3 fatty acid known for its excellent nutritional value and cholesterol lowering ability [4]. Additionally, flaxseed hulls are rich in lignans, containing about 9 to 30 mg/g, which is 40 to 800 times more than the level in 70 other kinds of lignan-containing plants, making flax the richest known source of lignans [5]. In recent years, lignans have received increasing attention for their unique functions, particularly in regulating and balancing estrogen levels in mammals [6]. Lignans have anti-cancer and antioxidant effects, including in the prevention of breast cancer [7,8,9,10]. Moreover, there has been extensive research on the extraction and purification of lignans, as well as on the detection of the physiological functions of metabolites [11,12,13]. Lignans are widely used in disease prevention and treatment, food production and processing, and industrial extraction methods.

Lignans are widely distributed in the roots, stems, leaves, and fruits of many plants. They play various roles, including contributing to plant resistance, environmental adaptation, and pest-related cytotoxicity resistance [14]. Currently, most plant lignans are not extensively studied due to their low content or limited economic benefits. However, some have gained importance due to their unique properties. For example, the well-known antitumor drug, podophyllotoxin, originates from lignans of the genus *Podophyllum* in the family Berberidaceae. Despite their low contents in plants, advancements in chemical synthesis and synthetic biology are gradually increasing their significance [15]. Lignans have been applied in various health fields, and they hold important positions among the many phenolic compounds studied in health. Specifically, (-)-secoisolariciresinol (SECO), an open-chain lignan found in flax, has been extensively studied due to its high content and role as a precursor to mammalian lignans.

Lignans can be classified as plant lignans or animal lignans based on their origin. In flax, SECO accounts for more than 95% of the lignan content [16,17]. Other lignans include matairesinol (MAT), pinoresinol (PINO), sesamin (Ses), lariciresinol (LARI), larch resinol, 1 ariciresinol (LCS), syringaresinol, isolariciresinol, arctigenin, nordihydroguaiaretic acid, and lilac. SECO and MAT are the most abundant lignans in flax, PINO in sesame, and PINO and LCS in *Brassica* vegetables [18]. The lignan contents of some plants are shown in Table 1.

## 2. The Basic Types of Lignans

**Figure 1:**
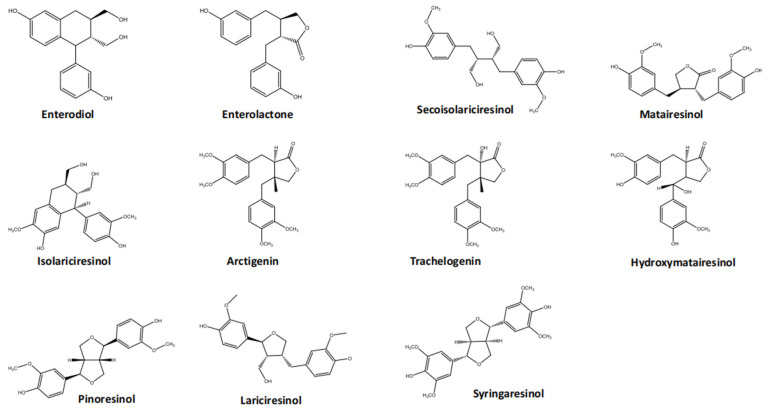
Example compounds of lignans in plants. PINO, SYRI, LARI, and Ses are furans, MAT is a dibenzyl butyrolactone, SECO is a 9,9′-dihydroxy dibenzylbutane, and isolariciresinol is a 9,9′-dihydroxyaryltetrahydronaphthalene.

Lignans are widespread throughout the plant kingdom and are found in almost all vascular plants. They are synthesized from mangiferous acid pathway initiation, with the phenylalanine pathway leading to the common lignan precursor, PINO. This precursor is then catalyzed by different enzymes along various pathways to produce lignans having different structures, types, and functions. Although lignans typically contain two C6-C3 skeletons linked by C8, newly derived lignans exhibit a rich variety of structures, with linkages occurring at other carbon positions besides C8 [19]. Additionally, different substitution patterns on the aromatic ring of the backbone and varying degrees of side chain oxidation contribute to the diversity of lignans, making them a significant class of secondary metabolites [16,20]. For clarity and classification, lignans are divided into eight subtypes: dibenzyl butyrolactone, dibenzyl cyclooctadiene, dibenzyl butane, aryl naphthalene, aryl tetrahydronaphthalene, furan, and furanone (Figure 1, only showing the example compounds from the 4 subtypes). This classification is based on the carbon skeleton, the manner in which oxygen binds to the skeleton, and the cyclization mode [16,19,21,22]. PINO, SYRI, LARI, and Ses are furans, MAT is a dibenzyl butyrolactone, SECO is a 9,9′-dihydroxy dibenzylbutane, and isolariciresinol is a 9,9′-dihydroxyaryltetrahydronaphthalene [23,24]. Each lignan subtype has its own unique and advantageous physiological activities. Among these, dibenzylbutyrolactone lignans are particularly noteworthy, because they are widely distributed in seed plants and found in nearly 200 species [23]. They are usually present as free glycosides or glycosides. In flax, typical representatives of this type of lignan include enterodiol, enterolactone, secoisolariciresinol, matiresinol, isoarctigenin, arctigenin, trachelogenin, hydroxymatairesinol, pinoresinol, lariciresinol, and syringaresinol [25]. Although these lignans are not abundant in flax, they have important physiological activities. MAT exhibits anti-leukemia properties by inhibiting DNA, RNA, and protein synthesis in leukemic cells [26]. Arctigenin has antiviral effects, inhibiting human immunodeficiency virus-1 replication and preventing the integration of viral DNA into the cellular genome [27].

## 3. Lignan Synthesis and Regulation

The synthesis of most secondary metabolites in plants begins in the chloroplast, where the metabolite erythrose-4-phosphate from the pentose phosphate pathway condenses with phosphoenolpyruvate, an intermediate product of glycolysis, to form chorismate. Chorismate is then converted to prephenic acid by chorismate mutase. Prephenic acid is further catalyzed by prephenate aminotransferase to produce arogenic acid, which is then converted to phenylalanine (Phe) by arogenate dehydratase. Among these three catalytic enzymes, arogenate dehydratase is considered the key enzyme, and its activity is usually inhibited by feedback from the Phe product. This pathway, known as the shikimic acid pathway, occurs entirely in the plastid [28,29,30,31,32]. This pathway produces both Phe and tyrosine, which are then transferred from the plastid to the cytoplasm. There, they contribute to the synthesis of plant secondary metabolites such as lignans, anthocyanins, and flavonoids [31].

The synthesis of lignans from Phe involves a multi-step reaction catalyzed by various enzymes. The process begins with the deamination of Phe by Phe ammonia-lyase to produce cinnamic acid. Cinnamic acid is then oxidized by cinnamate 4-hydroxylase to produce *p*-coumaric acid, which undergoes further oxidation to produce caffeic acid. Caffeic acid is then methylated by caffeic acid O-methyltransferase to form ferulic acid. Ferulic acid is converted to its activated form, feruloyl-CoA, by 4-coumarate-CoA ligase. Feruloyl-CoA is reduced to coniferyl aldehyde by cinnamoyl-CoA reductase. Finally, coniferyl aldehyde is reduced to coniferyl alcohol by cinnamyl-alcohol dehydrogenase and sinapyl alcohol dehydrogenase [33,34,35].

After the synthesis of PINO, the dirigent (DIR) guide protein couples two molecules of PINO in a specific stereostructure to produce one molecule of pineol. The synthesis of lignans from PINO can proceed via two different pathways. In one pathway, the furan structure of PINO is reduced to produce dibenzylbutane lignans, such as the linseed lignan SECO. In the other pathway, the furan structure of pineol remains intact, resulting in the production of methylenedioxy-bridged furanone lignans, such as the sesquiterpene lignan Ses [16,33,35,36]. In flax, the turpentine phenol is first reduced by PINO–LARI reductases (PLRs), resulting in the complete conversion to LARI [37], and the further catalysis by PLRs produces SECO [18,35,38]. Most secondary metabolites in plants exist in stable glycosylated forms. This glycosylation process is primarily mediated by the UGT family of enzymes, and, in flax, it is mainly catalyzed by UGT74S1 [39,40].

**Figure 2:**
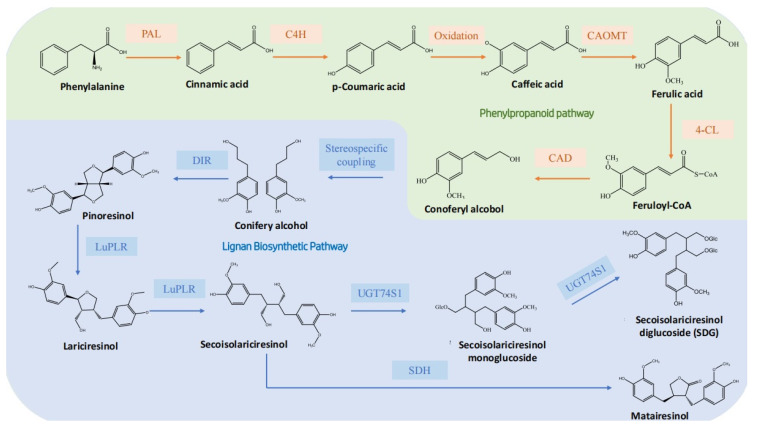
Lignan synthesis and regulation. The text on the green background represents the phenylpropanoid biosynthesis pathway. The text on the blue background represents the lignan biosynthetic pathway.

There are three rate-limiting steps in the synthesis of SECO, and each is catalyzed by three key enzymes, including stereoselective coupling by DIR-guided proteins, reduction by PLR, and glycosylation by UGT enzymes to convert PINO in flaxseed into the water-insoluble and stable SDG (Figure 2).

The DIR family was first studied in forsythia in 1990, in which it is involved in lignan biosynthesis [41]. Among the C6-C3 units synthesized by the Phe pathway, only pinealol is dimerized in a stereospecific manner, with the DIR-directing protein playing a key role in this process [42]. DIR family members encode both (−) and (+) PINO. The DIR encoding (−) PINO is expressed early in flaxseed coat development, generating (+) SECO, which is then used by UGT74S1 to catalyze the formation of (+) SDG. In contrast, the DIR encoding (+) PINO is expressed later in plant stem and leaf development, generating (−) SECO, which cannot form SDG [43]. Therefore, SDG is only detected in seeds [44].

Six potential DIR enzymes involved in the lignan synthesis pathway, LuDIR1–6, were further investigated. LuDIR1 expression in plant stems and leaves corresponds with the production of (+) PINO, whereas LuDIR5 and LuDIR6 are consistent with SDG accumulation in the seed coat, with LuDIR5 playing the primary role [16,43]. Due to the critical role of DIR proteins in lignan biosynthesis, studies have explored their application in the biosynthesis of the well-known antitumor lignan podophyllotoxin [15,45].

The PLRs catalyze the conversion of PINO to SECO. Two enantioselective PLR enzymes, LuPLR1 and LuPLR2, are present in flax. LuPLR1 is associated with the location and timing of SDG accumulation in the seed coat [46]. Moreover, the LuPLR1 promoter contains a binding region for the phytohormone abscisic acid (ABA), which promotes the transcriptional activation of LuPLR1. The addition of exogenous ABA enhances LuPLR1 transcription and facilitates the accumulation of SDG [44,47]. These results suggest that LuPLR1, with its enzyme activity and transcriptional expression regulated by ABA, is involved in the biosynthesis of lignans that promote SDG accumulation. LuPLR1 is mainly present in the seed coat, whereas LuPLR1 and LuPLR2 can be detected in plant roots and seeds. However, LuPLR2 is only expressed in stems and leaves, indicating that the abundant accumulation of SDG in seeds is not catalyzed by LuPLR2 [48]. LuPLR2 promotes the accumulation of yatein, which may be related to plant defense [37,44,46,49,50].

In the final step of SDG synthesis, SECO is catalyzed by the GT family to form a glycosylated structure, which is then linked to hydroxymethylglutaric acid to form an SDG–HMG complex that is stored in the flaxseed shell [43,51]. The plant GT family members are uridine glycosyltransferases (UGTs), which have the main role of catalyzing the transfer of activated UDP glycosides to specific receptor molecules [52,53,54]. This process increases the complexity and diversity of phytochemical structures, while also ensuring the stability and water solubility of plant natural products [55]. In flax, 137 *UGT* genes have been identified [40]. Further studies identified five UGT family enzymes, UGT74S1, UGT74T1, UGT89B3, UGT94H1, and UGT712B1 as having conserved sequences. Among them, UGT74S1 and UGT94H1 are highly expressed during seed development and are co-expressed with PLR, a key enzyme for SECO synthesis. The expression patterns of these genes are correlated with SDG biosynthesis, but UGT74S1 is the only enzyme that catalyzes the formation of SECO monoglucoside from SECO and further catalyzes the production of SDG from SECO monoglucoside [39,40,51]. Another study showed that mutating UGT74S1 prevents SECO from forming SDG, further demonstrating that UGT74S1 is the only enzyme responsible for catalyzing the glycosylation of SECO into SDG in flax [56].

## 4. Lignan Metabolic Pathways

**Figure 3:**
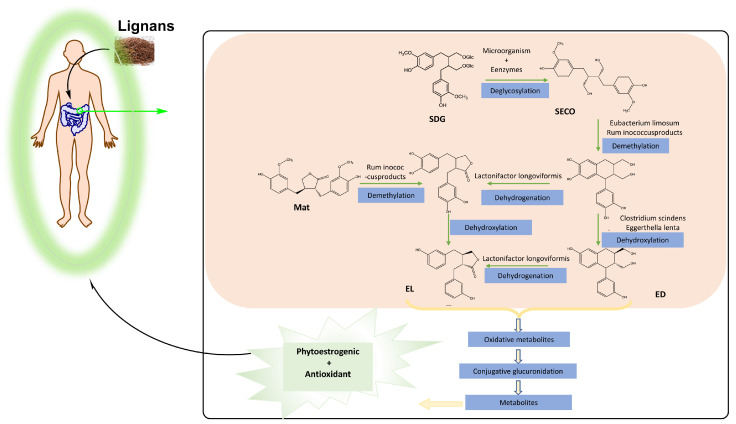
Lignan metabolic processes in humans.

After ingestion by animals, lignans from plants are first hydrolyzed in the gastrointestinal tract. This process releases SDG from its complex, converting it to SECO, which undergoes O-demethylation, dehydroxylation, and dehydrogenation reactions, primarily by bacteria in the colon, to produce enterolactone (EL). Enterodiol (EN) and EL were first isolated from human and animal serum, intestine, and bile samples in the early 1980s [57,58]. Due to their estrogenic and anti-estrogenic activities in humans and their plant origin, EN and EL are classified as phytoestrogens. Because they are produced in the intestines of animals, they are also referred to as mammalian lignans or enterolignans. Not only SECO, but also MAT, ART, syringaresinol, PDG, and Ses can undergo a series of intestinal reactions to eventually produce EL. However, due to lower levels and the higher number of required reactions, these lignans are significantly less utilized compared with the flax lignan SDG. Approximately 100% LARI, 72% SECO, and 55% PINO are metabolized to produce EN, whereas 62% MAT and EN are metabolized to EN [23,59,60,61,62,63]. The formation of these animal lignans plays an important physiological role in the body. The health benefits of flaxseed consumption are largely derived from EL and its oxidation products [64,65,66,67]. ED and EL are mainly absorbed in the colon. The key bacterial groups involved in converting plant lignans to animal lignans in the intestine have been identified [68,69,70,71]. Their identification has not only pinpointed the main contributors to the conversion process but has also enabled the in vitro production of animal lignans [70,72]. The deglycosylation of SDG is mainly carried out by bacteria, such as *Bacteroides distasonis*, *Bacteroides fragilis*, and *Bacteroides ovatus*, to produce SECO [59]. SECO is further demethylated by *Butyribacterium methylotrophicum*, *Eubacterium callanderi*, and other strains to produce dihydroxyenterodiol (2,3-bis (3,4-dihydroxybenzyl)-1,4-butanediol). *Clostridium scindens* and *Eggerthella lenta* then dehydroxylate SECO to produce EN, which is finally converted to EL by *Lactococcus* spp., *Ruminococcus* spp., and others [61]. Through these conversion steps, plant lignans, like SDG, are successfully converted into animal lignans, like EL. EL is then absorbed by intestinal cells, conjugated with glucosinolates or sulfates, and transported to the liver where it undergoes two stages of metabolism. Some metabolites partly return to the intestine via the hepatic intestinal circulation, while others circulate through the body via the bloodstream to exert physiological effects (Figure 3) [6,73].

## 5. Lignan Extraction, Isolation, Purification, and Toxicity

Lignans with various benefits have been applied in the food, clinical, and medical fields. Compared with chemical synthesis, the direct extraction of lignans from flax offers advantages, such as higher returns, simplicity, and cost-effectiveness. Consequently, several studies have focused on extracting and isolating flax lignans to achieve higher yields and product purity levels [12,18,74,75,76,77,78,79,80]. The extraction of lignans mainly refers to the flax lignan SDG, which is first released from its complex with HMG during the extraction process [81]. Because SDG is mainly located in the shell of the encapsulated flaxseed, accounting for 22.6% of the total seed weight, some studies have used flaxseed shells directly for extraction to achieve higher purity lignans [75,77,82]. To obtain higher purity lignans, whole flaxseed or defatted flaxseed powder have been used for extraction [83,84,85]. After grinding, the samples are first degreased using organic solvents like hexane, because the oil in the seeds can affect subsequent treatments [86]. The lignans are then extracted using organic solvents, typically ethanol, and supercritical fluid extraction and ultrasonic-assisted extraction methods to improve efficiency [4,75,84,85,87]. Separation, quantification, and identification are usually carried out using high-performance liquid chromatography, chromatography-tandem mass spectrometry, nuclear magnetic resonance spectrometry, and other techniques [75,79,83,88,89]. In recent years, several methods have been used to extract lignans, with the yield and purity of the products varying in method-dependent manners. The yield and purity of SDG depend significantly on the method and solvent used during extraction. Strong acids can break ester and glycosidic bonds, producing deglycosylated SDG or SECO, whereas alkaline hydrolysis only breaks ester bonds. Therefore, current extraction methods usually use alkaline hydrolysis to obtain intact SDG [75,83]. In some studies, the extraction methods were optimized by refining various conditions to obtain the highest lignan yield. The highest reported yield is 23.3 mg/g under optimal conditions [75].

Flaxseed contains excellent bioactive substances and has multiple health benefits, such as the ability to prevent and treat various diseases including cancer. Flaxseed also contains potential toxic substances and anti-nutritional factors, with the main toxins being multifaceted [90]. In order to improve nutritional and functional characteristics, advanced technologies are needed to remove the toxins without affecting their nutritional value, such as by inhibiting the genes that produce the toxins. The safety of flaxseed is still controversial, especially when consumed frequently in large quantities. In addition, the content of cyanogenic glycosides will increase due to the concentration effect of oil removal, and high levels of cyanogenic glycosides will limit the use of flaxseed powder as animal feed and its sales in various markets. Flaxseed protein is a prominent active compound with excellent health benefits. However, there are still some unresolved issues, such as the improvement of enzymatic hydrolysis conditions for bioactive peptides, clinical applications, and the elucidation of cellular toxicity mechanisms in some graded and purified structures [90]. Given the characteristics of peptides, including molecular weight, net charge, and hydrophobicity, it is necessary to develop improved purification processes by combining different separation methods, and to determine their roles and functions in living systems [91]. There is a lack of data on the toxicity caused by flaxseed oil, cyclic peptides, and polysaccharides. The dose-dependent toxicity of bioactive compounds from flaxseed (lignans, proteins, and oils) needs to be explored under different conditions and in different in vitro, animal, and human models [91,92]. To study the toxicity and cytotoxicity of flaxseed compounds, it is important to examine the role and function of flaxseed bioactive compounds in living systems. The role of these compounds in organisms, such as their functions, anti-tumor mechanisms, and applications, is not yet clear [92,93]. Flaxseed cyclic peptides have potential in the fields of affinity purification, fluorescence and antibody production, structural elucidation, and tumor activity relationships, and their effects on cell behavior need further exploration.

## 6. The Roles of Lignans in Human Health

After flaxseed consumption by humans and rats, SECO, mammalian lignans, intestinal lipids, and intestinal diols have been detected in urine, revealing significant increases in both blood and urinary excretions [72]. Lignans are metabolized by the human intestinal flora into EN and EL, which are absorbed through the intestinal tract, enter the hepatic intestinal circulation, and eventually reach the bloodstream, where they exert their physiological effects. The physiological activities of lignans in the body focus on reducing menopausal symptoms, heart disease, osteoporosis, and breast cancer risk [94]. For example, SDG can reduce the risk of breast cancer by delaying *N*-methyl-nitrosourea-induced tumorigenesis through the regulation of terminal differentiation [95].

Flax lignans exert their beneficial effects through intestinal lipids and metabolites that circulate to specific organs. Their physiological activity is primarily based on their estrogenic, anti-estrogenic, and antioxidant effects. For example, lignans exhibit strong antioxidative activities, functioning as hydroxyl radical scavengers. Recently, many new lignans with anti-inflammatory and antiviral activities have been discovered [96].

The health benefits of flaxseed are related to its high content of unsaturated fatty acids, which help reduce LDL-C, lower blood lipids, reduce blood viscosity, improve blood circulation, and prevent cardiovascular diseases. The lignans, particularly those related to podophyllotoxin, an important anti-cancer drug, have anti-cancer effects that are closely related to their phytoestrogenic and antioxidant properties [97].

**Figure 4:**
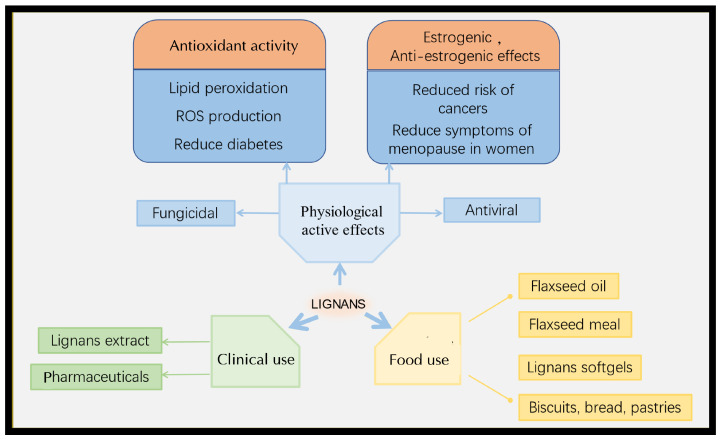
Functions of lignans in humans and their use.

Flax lignan consumption may prevent and reduce the risk of breast cancer, with varying effects observed before and after menopause. This difference may be related to hormonal changes, because estrogen promotes the occurrence and growth of breast cancer. After menopause, when estrogen levels decline, the preventive effect of lignan consumption appears more significant [98]. Phytoestrogen lignans are taken by mammals and converted into animal enterolignans, such as EL and EN. These enterolignans enter the bloodstream and compete with the animal estrogen estradiol for estrogen receptors α and β, thereby exerting preventive and delaying effects on hormone-sensitive tumors. These lignans exhibit both estrogenic and anti-estrogenic activities in vivo. Due to their structural similarity to estradiol, EL and EN compete for estrogen receptors in vivo, reducing the occurrence and development of breast cancer (Figure 4) [66,99,100].

Over the years, lignans have been extensively studied for their physiological activities, especially their antioxidative activities [101,102]. The antioxidative activity of SDG is expressed through its metabolites, ED and EL, which are effective in lowering serum cholesterol and reducing the development of atherosclerosis [101]. Consequently, the antioxidative activity of SDG significant contributes to delaying hypercholesterolemic atherosclerosis and diabetes. Additionally, high SDG intake reduces liver serum triglycerides and cholesterol, suggesting that lignans have lipid-lowering effects (Figure 4) [103].

## 7. Summary and Outlook

This paper discussed the discovery of lignans in plants and the subsequent studies on their extraction, optimization, synthetic pathways, metabolism, bioavailability, and anti-disease mechanisms. As living standards have improved, the disease-preventive physiological activities of lignans have garnered increased attention. Various lignans extracted and identified from different plants have shown clear antiviral and anti-inflammatory effects. The important antitumor lignans, onychotoxins, play important roles in medicine and have also prompted the industrial production of lignans using synthetic biology. SDG is the most abundant lignan in flax, and its synthesis pathway is well-studied. However, there is limited research on the transcription factors that regulate the key enzymes of the pathway, and the downstream pathways of SDG metabolism remain largely unexplored. While research has elevated SDG as the most utilized lignan, there is potential for downstream products having higher utilization values, as exemplified by podophyllotoxins. Therefore, identifying and utilizing key genes and downstream products in lignan synthesis represents a promising direction for future lignan research.

## Figures and Tables

**Table 1:** The lignans contents in different plants.

Plant Name	Different Lignans Content				Total Content	Methods	Reference
	SECO	MAT	PINO	LAR			
**Oilseeds and nuts**							
Flaxseed	294,210	553	3324	3041	301,129	μg/100 g fresh edible weight	[18]
Flaxseed	7208	0	2	29	-	μg/100 g wet basis	[19]
Flaxseed (whole)	11,845	26	383	220	-	μg/100 g wet basis	[18]
Flaxseed	369,900	1087	-	-	370,987	μg/100 g dry wt	[20]
Sunflower seeds	26.2	0.5	33.9	149.7	-	μg/100 g wet basis	[19]
Sesame seed	66	481	29,331	9470	39,348	μg/100 g fresh edible weight	[18]
**Vegetables**							
Garlic	50	0	200	286	536	μg/100 g fresh edible weight	[18]
Curly kale	19	12	1691	599	2321	μg/100 g fresh edible weight	[18]
Broccoli	38	0	315	972	1325	μg/100 g fresh edible weight	[18]
Asparagus	743	14	122	92	1034		[16]
Cabbages (Finnish database)	30.3	0.2	-	-	30.5	Mean of the group	[21]
Cabbages (Dutch database)	8	2	335	255	600	Weighted mean (most common cabbages)	[21]
Fruit vegetables (Finnish database)	5.49	0.01	-	-	5.5	Weighted mean (tomato and cucumber)	[21]
Fruit vegetables (Dutch database)	10	0	19	103	132	Mean of sweet pepper, zucchini, cucumber and tomato	[21]
Onion-family vegetables (Finnish database)	20	3.8	-	-	23.8	Mean, SECO-value weighted by onion	[21]
Onion-family vegetables (Dutch database)	34	0	100	153	287	Mean of garlic, leek and onion	[21]
**Fruits**							
Apricot	31	0	314	105	450	μg/100 g fresh edible weight	[18]
Strawberry	5	0	117	212	334	μg/100 g fresh edible weight	[18]
Peach	27	0	186	80	293	μg/100 g fresh edible weight	[18]
Yuzu	26	-	654	192	1291	μg/100 g wet basis	[16]
Valencia orange	56	-	51	193	521	μg/100 g wet basis	[16]
**Cereal and grain products**							
Rye (Finnish database)	40	55	-	-	95	Weighted mean (whole grain rye flour)	[21]
Rye (Dutch database)	23	20	246	175	458	Ryebread × 1.43	[21]
Wheat (Finnish database)	20	5	-	-	25	Weighted mean (wheat flour)	[21]
Wheat (Dutch database)	12	0	29	60	99	Wheatbread × 1.43	[21]
Rice (Finnish database)	26.4	1	-	-	27.4	Means of rice/rice-containing foods	[21]
Rice (Dutch database)	1.5	1	3.5	17.5	23.5	Means of white rice and whole grain rice	[21]
Pasta and macaroni (Finnish database)	6.8	0.4	-	-	7.2	Mean of all pastas	[21]
Pasta and macaroni (Dutch database)	18	5	0	4	9	Cooked pasta	[21]
